# Is Spectral-Domain Optical Coherence Tomography Essential for Flexible Treatment Regimens with Ranibizumab for Neovascular Age-Related Macular Degeneration?

**DOI:** 10.1155/2013/786107

**Published:** 2013-11-11

**Authors:** Abdullah Ozkaya, Zeynep Alkin, Hande Mefkure Ozkaya, Alper Agca, Engin Bilge Ozgurhan, Yalcin Karakucuk, Ahmet Taylan Yazici, Ahmet Demirok

**Affiliations:** ^1^Beyoglu Eye Training and Research Hospital, Bereketzade Camii Sok., Kuledibi, Beyoglu, 34421 Istanbul, Turkey; ^2^Sisli Etfal Training and Research Hospital, 34360 Istanbul, Turkey; ^3^Department of Ophthalmology, Medeniyet University, 34772 Istanbul, Turkey

## Abstract

*Purpose*. To evaluate the ability of spectral-domain optical coherence tomography to detect subtle amounts of retinal fluid when the choroidal neovascularization is detected as inactive via time-domain optical coherence tomography and clinical examination in neovascular age-related macular degeneration (nAMD) patients. *Methods*. Forty-nine eyes of 49 patients with nAMD after ranibizumab treatment were included in this cross-sectional, prospective study. All patients were imaged with TD-OCT and SD-OCT at the same visit one month after a ranibizumab injection. The presence of subretinal, intraretinal, and subretinal pigment epithelium fluid (subRPE) in SD-OCT was evaluated; also mean central retinal thickness (CRT) and the rate of vitreoretinal surface disorders detected via the two devices were evaluated. *Results*. The mean CRT via TD-OCT and SD-OCT was 218.1 ± 51.3 and 325.7 ± 78.8 microns. Sixteen patients (32.6%) showed any kind of retinal fluid via SD-OCT. In detail, 8 patients (16.3%) showed subretinal fluid, 10 patients (20.4%) showed intraretinal fluid, and 3 patients (6.1%) showed SubRPE fluid. The ability of detecting vitreoretinal surface disorders was comparable between the two devices, except vitreomacular traction. *Conclusion*. SD-OCT is essential for the nAMD patients who are on an as-needed treatment regimen with ranibizumab. Only TD-OCT and clinical examination may cause insufficient treatment in this group of patients.

## 1. Introduction

Neovascular age-related macular degeneration (nAMD) is the leading cause of visual loss among the elderly population in developed countries [1, 2]. Before the introduction of intravitreal antivascular endothelial growth factor therapy for nAMD, only prevention for visual loss might have been achieved in a limited number of patients with different treatment options like laser photocoagulation and photodynamic therapy [3–7]. The introduction of bevacizumab (full length antibody against VEGF-A) and ranibizumab (Fab part of antibody against VEGF-A) has led the vast majority of the patients to prevent the baseline visual acuity [8–16]. The pivotal multicenter studies with ranibizumab, like MARINA, ANCHOR, PRONTO, EXCITE, and CATT, showed that ranibizumab is effective to prevent baseline visual acuity in up to 95% of the patients and is effective to make an improvement in visual acuity in up to 40% of the patients [9–13]. Monthly ranibizumab treatment for nAMD was found to be effective in MARINA and ANCHOR studies; however, an attempt then aroused to decrease the injection numbers. Therefore, studies like PRONTO and FUSION were designed to evaluate the effectiveness of as-needed treatment regimens, and ranibizumab was found to be effective in the treatment of nAMD on as-needed treatment regimens [11, 14]. CATT study was designed to compare the outcomes of monthly and as-needed treatment regimens for both bevacizumab and ranibizumab [13]. This study revealed that the visual acuity outcomes were similar for monthly bevacizumab versus monthly ranibizumab, monthly bevacizumab versus as-needed bevacizumab, and monthly ranibizumab versus as-needed ranibizumab. Only monthly ranibizumab was found to be more effective than as-needed bevacizumab. 

Optical coherence tomography (OCT) is a noninvasive diagnostic device that provides cross-sectional images of the retina like ultrasound [17, 18]. However, OCT uses light waves instead of sound. Time-domain OCT systems have a scan rate of 400 A-scans per second which allow an axial resolution of 8–10 microns, and spectral-domain OCT systems have a scan rate of 20.000–65.000 A-scans per second which allow an axial resolution of 3–7 microns and provide a three-dimensional view of the retina. Although fluorescence angiography is the gold standard for the diagnosis and followup of retinal vascular diseases and nAMD for several decades, it was largely replaced by OCT after the introduction of OCT-guided, flexible treatment regimens for nAMD with anti-VEGF agents [17, 18].

In this study, we aimed to evaluate the activity of choroidal neovascularization with SD-OCT in the nAMD patients who were on an as-needed treatment regimen with ranibizumab and were diagnosed to have an inactive choroidal neovascularization with TD-OCT and clinical examination one month after intravitreal ranibizumab injection.

## 2. Material and Methods

This prospective, comparative, cross-sectional study included 49 eyes of 49 consecutive nAMD patients who were on an as-needed treatment regimen with intravitreal ranibizumab (IVR). The patients underwent IVR injections at Beyoglu Eye Training and Research Hospital between March 2012 and April 2012. Approval for data collection and analysis was obtained from the ethics committee of the hospital, and written informed consent was provided from all patients. The methodology of the study was designed in accordance with the tenets of the Helsinki Declaration. 

The treatment regimen was as follows: for the first three months of treatment, all patients received monthly doses of ranibizumab 0.5 mg/0.05 mL. The patients were then examined monthly and were retreated if they met any of the following criteria:

- visual loss of 1 or more lines,
- new perimacular hemorrhage,
- evidence of CNV enlargement on examination or fluorescein angiography,
- any amount of persistent subretinal, intraretinal, or subretinal pigment epithelial (SubRPE) fluid one month after an injection.

The patients who had a diagnosis of nAMD, were currently on an as-needed treatment regimen with IVR, underwent an IVR injection a month ago, did not experience visual loss since the last visit, did not show any activity of CNV on fundus examination, and did not show any amount of subretinal, intraretinal, or subretinal pigment epithelium (SubRPE) fluid on TD-OCT were included in the study. The patients were not included in the study if the quality of the OCT scans were not sufficient with any of the TD-OCT or SD-OCT devices.

Data collected from the patients' records included age, gender, central retinal thickness defined by TD-OCT and SD-OCT, the fluid type revealed by SD-OCT, and the vitreoretinal surface abnormalities revealed by TD-OCT and SD-OCT.

All patients underwent a standardized examination including measurement of BCVA via the early treatment diabetic retinopathy study (ETDRS) chart at 4 meters, slit-lamp biomicroscopy and fundus examination, and measurement of intraocular pressure (IOP) via applanation tonometry. Fundus photography, and optical coherence tomography (OCT) imaging with TD-OCT (Stratus OCT TM; Carl Zeiss Meditec Inc., Dublin, CA, USA) and SD-OCT (Spectralis; Heidelberg Engineering, Heidelberg, Germany) were performed. All the OCT images were assessed by the same physician (AO). 

Time-domain OCT imaging was performed via Stratus OCT 3000 (Carl Zeiss, Meditec Inc., Dublin, CA, USA). Fast macular thickness map (FastMac) protocol was used to obtain the retinal scans, within a scan time of 1.9 seconds, which acquires six evenly spaced 6-millimeter (mm) radial lines, each consisting of 128 A scans per line, intersecting at the fovea (total of 768 sampled points). The CRT was defined as the distance between the internal limiting membrane (ILM) and the photoreceptor junction of the inner and outer segments via the automatic analysis package of Stratus OCT. 

Spectral-domain OCT imaging was performed via Spectralis OCT (Heidelberg Engineering, Heidelberg, Germany). Fast macula protocol was used to obtain the retinal scan, with a automatic real time (ART) mean value of 9, which acquires 25 horizontal lines (6 × 6 mm area), each consisting of 1024 A scans per line. The CRT was defined as the distance between the ILM to the outer border of the retinal pigment epithelium via the automatic segmentation algorithms of the Spectralis software.

The main outcome measure of the study was the rate of the patients who had any amount of subretinal, intraretinal, or SubRPE fluid on SD-OCT and who were diagnosed as inactive after clinical examination and TD-OCT imaging. The secondary outcome measures were the CRT values defined via the two devices and the ability of detecting vitreoretinal surface disorders via the two devices.

For the statistical analyses, the mean (SD) of the differences was calculated. Independent sample *t*-test was used to compare continuous parameters between the two devices, and chi-square test was used for nominal parameters. Statistical analyses were made using commercially available software SPSS version 16.0 (SPSS Inc., Chicago, IL). *P* value of 0.05 was considered statistically significant.

## 3. Results

Overall mean age was 72.6 ± 8.8 years (range 56–86 years). 29 patients (59.2%) were female and 20 patients (40.8%) were male. Mean CRT of the patients via TD-OCT was 218.1 ± 51.3 microns (range 139–418 microns), and the mean CRT via SD-OCT was 325.7 ± 78.8 (range 222–508 microns). The mean ratio between the mean CRT measured with SD-OCT and CRT measured with TD-OCT was 1.51 ± 0.09. 

**Figure 1:**
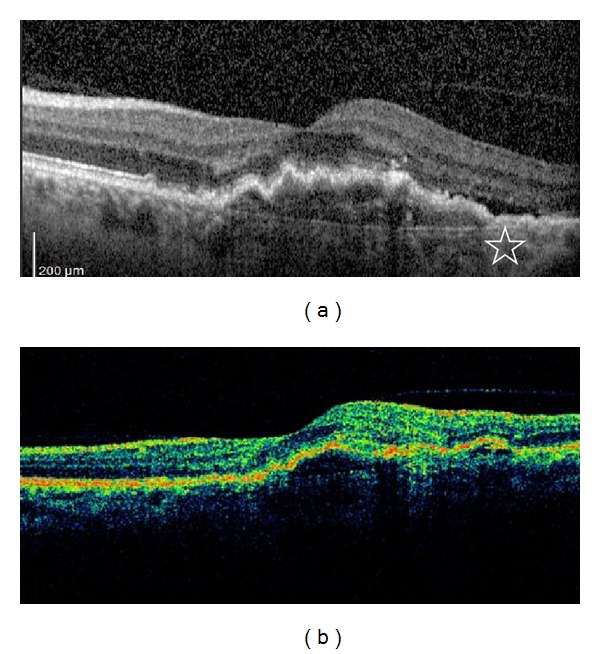
Spectral-domain optical coherence tomography (a) and time-domain optical coherence tomography (b) scans of a patient; the white star shows the subretinal fluid on spectral-domain optical coherence tomography.

As the patients who did not show any amount of subretinal, intraretinal, or subRPE fluid in TD-OCT were the subjects of this study, any kind of retinal fluid was not detected in TD-OCT. However, 16 of the 49 patients (32.6%) showed subretinal, and/or intraretinal, and/or subRPE fluid on SD-OCT. In detail, 8 patients showed (16.3%) subretinal fluid, 10 patients (20.4%) showed intraretinal fluid, and 3 patients (6.1%) showed SubRPE fluid (Table 1) (Figure 1).

The ability of the two devices in detecting vitreoretinal surface abnormalities was summarized in Table 2.

## 4. Discussion

Since the introduction of the SD-OCT, the resolution of retinal scans and parallelly our diagnostic ability increased. Many studies compared the TD-OCT and SD-OCT systems in regard to the fluid detection, and retinal thickness measurements [17–23]. In previous studies the, OCT devices were usually compared in patients having had nAMD, diabetic macular edema, and retinal vein occlusion [17–23]. However, these studies mostly evaluated the ability of these devices in detecting subretinal, intraretinal, or subRPE fluid and compared the CRT measurements between them [17–21]. In this study we have chosen a different methodology. We delineated the nAMD patients who were diagnosed to have an inactive CNV via the TD-OCT and clinical examination, and then we evaluated them with the SD-OCT in order to detect the additional sensitivity provided via the latter device. The additional sensitivity of the SD-OCT was 32.6% in regard to CNV activity.

In a study by Kakinoki et al., the macular thickness in diabetic macular edema measured with TD-OCT and SD-OCT were compared [17]. The macular thickness values were well correlated; however, the mean macular thickness detected with the SD-OCT was reported to be 45 microns thicker than the TD-OCT. Hatef et al. compared two different SD-OCT devices with TD-OCT in patients with macular edema secondary to diabetic retinopathy and retinal vein occlusion [18]. The macular thickness values detected with both SD-OCT devices were also found to be higher than the values detected with TD-OCT.

Eriksson et al. showed that SD-OCT increased the repeatability and decreased variability in nAMD patients, and demonstrated a significant advantage of SD-OCT over TD-OCT [19]. Krebs et al. mentioned the difference of CRT values detected with the TD-OCT and the SD-OCT devices and attributed this variability to the different posterior reference lines of the devices [20]. They advised avoiding using different devices during an ongoing study and suggested a formula for comparing the values defined by the two devices. In a case series by Luviano et al., SD-OCT was found to be superior to TD-OCT in regard to fluid and cyst detection [21]. 

Querques et al. compared the ability of the 2 SD-OCT and TD-OCT devices in detecting the disease activity in the nAMD patients [22]. The patients were evaluated at different time points after IVR injection. Cirrus SD-OCT (Cirrus HD-OCT, Carl Zeiss-Meditec, Inc., Dublin, CA) and Spectralis SD-OCT were found to be superior to TD-OCT especially in detecting SRF and PED. They also reported that the correcting factor should be 1.48 to extrapolate the CRT measurements from Stratus TD-OCT to Spectralis SD-OCT, which was comparable with our value of 1.51.

Cukras et al. reported that Cirrus SD-OCT was able to detect the presence of the exudative activity (subretinal or intraretinal fluid) in 48% of the nAMD patients who were defined as inactive with Stratus TD-OCT [23]. Sayanagi et al. compared 4 different SD-OCT devices with Stratus TD-OCT in regard to disease activity in the nAMD patients after intravitreal ranibizumab treatment [24]. They reported that all 4 SD-OCT devices were superior to TD-OCT in detecting subretinal fluid in three-dimensional cube mode. However, in linear B-scan mode, only the Spectralis SD-OCT was reported to be superior to the Stratus TD-OCT in detecting subretinal fluid, and the OCT-1000 was reported to be superior in detecting sub-RPE fluid. In addition, the presence of subretinal fluid, intraretinal cysts, and subRPE fluid on the Spectralis SD-OCT was reported to be 16%, 11%, and 13%, respectively, when TD-OCT did not show any sign of disease activity. These results were comparable to our study. Only the rate of the sub-RPE fluid detection of our study was lower than that of Sayanagi et al. 

The VA loss during the as-needed treatment regimens for the nAMD which is usually attributed to the treatment delays, the low injection numbers, the presence of PED at the baseline, and the CRT fluctuations during the treatment is usually irreversible [25–29]. Except one, three of these factors, treatment delays, CRT fluctuations, and low injection numbers are closely related with our ability to detect disease activity. Therefore, in the era of the SD-OCT devices, the TD-OCT seems inaccurate and insufficient in deciding whether or not retreatment is necessary at the monthly visits of the nAMD patients. On the other hand, while the CRT is an important prognostic factor for the diabetic macular edema and retinal vein occlusion patients, and TD-OCT is comparable with the SD-OCT devices in this group of patients, TD-OCT may still have a role in our retreatment decisions. 

Limitations of this study include the relatively small number of patients and the one-sided assessment between the two devices (we did not evaluate the nonactive patients via the SD-OCT with TD-OCT). Strengths of this study were the prospective design and the homogeneity of the patients.

In conclusion, the previous studies and the present study suggest that SD-OCT is essential in the followup of the nAMD patients who are under an as-needed treatment regimen. Using TD-OCT may result in worse visual outcomes due to the treatment delays and inadequate treatment.

## Figures and Tables

**Table 1:** The findings on spectral-domain optical coherence tomography when time-domain optical coherence tomography and clinical examination showed no sign of choroidal neovascularization activation.

Variables	Absence on TD-OCT	
	Presence on SD-OCT	Absence on SD-OCT
Subretinal fluid	8 patients (16.3%)	41 patients (83.7%)
Intraretinal cysts, or fluid	10 patients (20.4%)	39 patients (79.6%)
SubRPE fluid	3 patients (6.1%)	46 patients (93.9%)

TD-OCT: time-domain optical coherence tomography; SD-OCT: spectral-domain optical coherence tomography; SubRPE: subretinal pigment epithelium.

**Table 2:** The ability of spectral-domain optical coherence tomography and time-domain optical coherence tomography to detect the vitreoretinal surface disorders.

Variables	TD-OCT	SD-OCT	*P *
ERM	7 patients	9 patients	0.7
PVD	2 patients	4 patients	0.4
VMT	1 patients	4 patients	0.19

ERM: epiretinal membrane; PVD: posterior vitreous detachment; VMT: vitreomacular traction, TD-OCT: time-domain optical coherence tomography; SD-OCT: spectral-domain optical coherence tomography; *P*: *P* value, chi-square test.
